# Decoding the Complexity of Systemic Inflammation Predictors in Locally Advanced Cervical Cancer, with Hemoglobin as the Hidden Key (the ESTHER Study)

**DOI:** 10.3390/cancers15205056

**Published:** 2023-10-19

**Authors:** Federica Medici, Martina Ferioli, Ludovica Forlani, Viola Laghi, Johnny Ma, Savino Cilla, Milly Buwenge, Gabriella Macchia, Francesco Deodato, Maria Vadalà, Claudio Malizia, Luca Tagliaferri, Anna Myriam Perrone, Pierandrea De Iaco, Lidia Strigari, Alessandra Arcelli, Alessio Giuseppe Morganti

**Affiliations:** 1 Radiation Oncology, Department of Medical and Surgical Sciences-DIMEC, Alma Mater Studiorum University of Bologna, 40138 Bologna, Italy; martina.ferioli4@unibo.it (M.F.); ludovica.forlani@studio.unibo.it (L.F.); viola.laghi@studio.unibo.it (V.L.); johnny.ma@studio.unibo.it (J.M.); milly.buwenge2@unibo.it (M.B.); myriam.perrone@aosp.bo.it (A.M.P.); pierandrea.deiaco@unibo.it (P.D.I.); alessandra.arcelli@aosp.bo.it (A.A.); alessio.morganti2@unibo.it (A.G.M.); 2 Radiation Oncology, IRCCS Azienda Ospedaliero-Universitaria di Bologna, 40138 Bologna, Italy; 3 Medical Physics Unit, Gemelli Molise Hospital-Università Cattolica del Sacro Cuore, 86100 Campobasso, Italy; savinocilla@gmail.com; 4 Radiotherapy Unit, Gemelli Molise Hospital, Fondazione Policlinico Universitario A. Gemelli, IRCCS, 86100 Campobasso, Italy; gabriella.macchia@gemellimolise.it (G.M.); francesco.deodato@unicatt.it (F.D.); 5 Nuclear Medicine, IRCCS Azienda Ospedaliero-Universitaria di Bologna, 40138 Bologna, Italy; maria.vadala@aosp.bo.it (M.V.); claudio.malizia@aosp.bo.it (C.M.); 6 UOC di Radioterapia Oncologica, Dipartimento Diagnostica per Immagini, Radioterapia Oncologica ed Ematologia, Fondazione Policlinico Universitario Agostino Gemelli IRCCS, 00168 Roma, Italy; luca.tagliaferri@policlinicogemelli.it; 7 Division of Gynecologic Oncology, IRCCS Azienda Ospedaliero-Universitaria di Bologna, 40138 Bologna, Italy; 8 Medical Physics, IRCCS Azienda Ospedaliero-Universitaria di Bologna, 40138 Bologna, Italy; lidia.strigari@aosp.bo.it

**Keywords:** anemia, brachytherapy, chemoradiation, cervical cancer, hemoglobin, inflammatory index, overall survival, observational study, prognostic factor, predictive model

## Abstract

**Simple Summary:**

We explored whether specific factors, like inflammation indicators in the blood, could help predict treatment outcomes for locally advanced cervical cancer (LACC). LACC is generally treated with a combination of chemotherapy and radiation. We wanted to see if these factors could help physicians personalize treatments for better results. Our study involved looking at various aspects, including inflammation indices in the blood and various clinical treatment details, in LACC patients. While some factors, such as age and hemoglobin levels, seemed to predict outcomes, there was no clear connection between inflammation indicators in the blood and results. These findings challenge previous ideas and highlight the importance of considering multiple factors to predict the prognoses of LACC patients.

**Abstract:**

Locally advanced cervical cancer (LACC) is treated with concurrent chemoradiation (CRT). Predictive models could improve the outcome through treatment personalization. Several factors influence prognosis in LACC, but the role of systemic inflammation indices (IIs) is unclear. This study aims to assess the correlation between IIs and prognosis in a large patient cohort considering several clinical data. We retrospectively analyzed pretreatment IIs (NLR, PLR, MLR, SII, LLR, COP-NLR, APRI, ALRI, SIRI, and ANRI) in 173 LACC patients. Patient, tumor, and treatment characteristics were also considered. Univariate and multivariate Cox’s regressions were conducted to assess associations between IIs and clinical factors with local control (LC), distant metastasis-free survival (DMFS), disease-free survival (DFS), and overall survival (OS). Univariate analysis showed significant correlations between age, HB levels, tumor stage, FIGO stage, and CRT dose with survival outcomes. Specific pretreatment IIs (NLR, PLR, APRI, ANRI, and COP-NLR) demonstrated associations only with LC. The multivariate analysis confirmed Hb levels, CRT dose, and age as significant predictors of OS, while no II was correlated with any clinical outcome. The study findings contradict some prior research on IIs in LACC, emphasizing the need for comprehensive assessments of potential confounding variables.

## 1. Introduction

Cervical cancer stands as a prevalent global malignancy [1]. Locally advanced cervical cancer (LACC) patients are commonly treated with concurrent chemoradiation (CRT), based on the simultaneous administration of chemotherapy (CHT) and radiotherapy (RT), and represent the standard therapeutic approach. Though CRT proves effective in achieving substantial local tumor control [2], a notable subset of patients, around one third, experience treatment failure [3,4]. Over the past years, interest in oncology has gradually grown in the development of predictive models of outcome. In fact, predictive models can offer the potential for clinicians to foresee clinical outcomes following specific treatments, thereby enabling personalized medical interventions based on individual stages, recurrence risks, and demographic attributes.

Several predictive factors have been scrutinized and incorporated into these models, specifically within the context of LACC. Elements such as tumor size, histological type, lymph node metastases, and Federation of Gynecology and Obstetrics (FIGO) stage have been identified as significant prognosticators linked to overall survival (OS) [5,6]. Moreover, anemia has long been acknowledged as an unfavorable prognostic determinant among LACC patients [7,8,9,10].

To enhance the precision of outcome predictions, thereby enabling treatment tailoring according to prognostic profiles, recent studies have explored the predictive utility of different systemic inflammation indices (IIs) [11]. These indices have demonstrated a substantial correlation with treatment outcomes also in patients with LACC [11,12,13,14,15,16,17,18,19,20,21,22,23,24,25,26,27]. Specifically, investigations involving LACC patients showed a significant impact of IIs on tumor response [25], disease-free survival (DFS) [13,15,17,18,19,22,26,27], and OS [15,16,17,18,19,20,22,24,26,27].

However, many of these studies have primarily focused on a single index [11,13,16,18,20,26,27] or a limited array of indices [12,14,15,19,23,25], often without a thorough assessment of potential confounding variables [13,16,17,22,23,25,26]. Therefore, the principal objective of this study is to comprehensively analyze the predictive capabilities of a spectrum of systemic IIs within an extensive cohort of LACC patients. This analysis will incorporate pertinent clinical prognostic factors, encompassing clinical, tumor-related, and treatment-related data. The final aim of this study is to evaluate whether the prognosis of LACC patients can be improved by modulating the treatment based on the values of the IIs.

## 2. Materials and Methods

### 2.1. Study Objective and Design

This study aimed to investigate how various systemic IIs are linked to the prognosis of LACC by examining their effects on key outcomes: local control (LC), distant metastasis-free survival (DMFS), DFS, and OS.

More precisely, our main objective in this analysis was to externally validate the predictive significance of the IIs’ pretreatment values, as well as the associated thresholds proposed in the existing literature, within the context of LACC [11,12,13,14,15,16,17,18,19,20,21,22,23,24,25,26,27]. Additionally, we pursued a secondary goal of conducting an exploratory assessment regarding the predictive influence of IIs’ values observed at the end of CRT, considering the relatively limited information available on the impact of post-treatment IIs’ values [15]. Lastly, our aim was to assess whether the differences between pre- and post-treatment values (Delta-indexes) showed significant correlations with the outcomes under examination. Also, this evaluation was conducted due to the limited data existing in the literature on this aspect [15,23].

We conducted a single-center retrospective analysis of patients treated at our institution between July 2007 and July 2021. These patients were part of an approved observational study (ESTHER study, code CE 973/2020/Oss/AOUBo) overseen by our local Ethical Committee. All patients provided informed consent to participate, and no exclusions were made to maintain the study’s real-world applicability. However, patients were excluded from this analysis in case of treatment performed with palliative intent and if essential data from clinical records were unavailable. This includes situations where necessary blood-test results for calculating the IIs were missing.

### 2.2. Staging, Treatment, and Follow-Up

The retrospective classification of LACCs was based on the 2018 FIGO staging system [28]. Patients underwent definitive concurrent CRT, which involved a combination of external beam RT (EBRT) targeting the pelvic area (doses of 45–50 Gy, delivered in fractions of 1.8–2 Gy) and intracavitary interventional RT (brachytherapy—BRT), administered as either pulsed or high dose rate. The goal was to achieve a total equivalent dose of 80–90 Gy for the gross tumor volume (GTV). The clinical target volume (CTV) encompassed the GTV, uterus, upper third of the vagina, parametria, and pelvic nodes (internal, external, common iliac, obturator, and presacral nodes), with a 7 mm expansion. Treatment of para-aortic lymph nodes was considered only if there were nodal metastases in that region. The planning target volume was defined as the CTV with an additional 10 mm expansion in all directions. Metastatic or suspicious pelvic nodes received an additional radiation boost in a sequential or simultaneously integrated timing, reaching a total equivalent dose of 55–65 Gy. Patient alignment was monitored daily using electronic portal imaging devices until 2015 and then shifted to onboard cone-beam CT [29]. Concurrent CHT involved intravenous Cisplatin (40 mg/m^2^ weekly). Patients received follow-up through physical examinations every three months for two years and subsequently every six months for three years. Thoracic–abdominal–pelvic computed tomography scans were performed as needed clinically or every six months during the first two years and annually in the subsequent three years.

### 2.3. Examined Parameters

#### 2.3.1. Patient, Tumor, and Treatment Information

This analysis encompassed patient-related details such as age and hemoglobin (Hb) level, measured in g/100 mL). Additionally, we considered tumor-related information, including histological type, FIGO stage, clinical tumor stage, clinical nodal stage, and maximum tumor diameter. Moreover, treatment-related data comprised RT technique, EBRT dose and fractionation applied to the pelvic region, BRT boost dose, total tumor dose, and overall treatment time (including both EBRT and BRT, measured in days).

#### 2.3.2. Inflammation Indices

The analysis included the examination of various IIs, including neutrophil-to-lymphocyte ratio (NLR), platelet-to-lymphocyte ratio (PLR), monocyte-to-lymphocyte ratio (MLR), systemic immune inflammation index (SII, calculated as the product of platelet count and neutrophil count divided by lymphocyte count), leukocyte-to-lymphocyte ratio (LLR), a combination of platelet (PLT) count and NLR (COP-NLR), which was categorized as follows: 0 for NLR < 3 and PLT < 300 × 10^9^/L, 1 for NLR > 3 or PLT > 300 × 10^9^/L, and 2 for NLR > 3 and PLT > 300 × 10^9^/L. Additionally, the analysis included the aspartate amino-transferase/platelet count ratio index (APRI), calculated as [aspartate aminotransferase (IU/L) divided by upper limit normal/platelet count (×10^9^/L)] multiplied by 100, the aspartate aminotransferase-to-lymphocyte ratio index (ALRI), calculated as aspartate aminotransferase value (U/L) divided by lymphocyte count (×10^9^/L), the systemic inflammatory response index (SIRI), calculated as neutrophil count multiplied by monocyte count divided by lymphocyte count, and, finally, the aspartate transaminase-to-neutrophil ratio index (ANRI), calculated as aspartate aminotransferase divided by neutrophil count.

Considering the primary aim of our analysis, which involves externally validating a range of IIs assessed in cases of LACC, along with the varying threshold values outlined in the existing literature, we undertook the process of dichotomizing the index-related data. Specifically, we utilized the published cut-off points, particularly focusing on those associated with significant clinical outcomes. Additionally, we conducted an exploratory analysis on the prognostic significance of the II values assessed at the conclusion of concurrent CRT and of pre-post-treatment variations of the indices (Delta-indices). In this scenario, we dichotomized the parameters using the median value, owing to the relatively limited availability of published data in this context.

### 2.4. Statistical Analysis

Patient and tumor characteristics, as well as treatment data, were depicted using descriptive statistical methods. Categorical data were presented using numbers and percentages, while continuous data were expressed in terms of medians and ranges. LC was computed as the time elapsed from the start of concurrent CRT until the evidence of local–regional recurrence, as identified through imaging studies or clinical observations, or until the last follow-up in patients without pelvic recurrence. DMFS was computed as the time span from the initiation of CRT until the occurrence of distant failure, detected through imaging studies or clinical observations, or until the last follow-up in patients without extrapelvic recurrence. DFS was calculated as the period from CRT initiation until any treatment failure or until the last follow-up in patients without a recurrence of LACC. OS was defined as the interval between CRT initiation and the time of death or the most recent follow-up date.

For each of these four endpoints, survival curves were generated using the Kaplan–Meier method, and a univariate analysis (log-rank) was carried out, encompassing all the specified variables. Furthermore, a multivariate Cox’s regression analysis was conducted, involving variables with a *p*-value of less than 0.1 in the univariate analysis. A significance level of 5% was employed (*p*-value < 0.05). If the assessment of various cut-off points for a particular II indicated statistical significance for only one specific cut-off, that particular value was exclusively integrated into the multivariate analysis. Moreover, when multiple cut-off values exhibited statistically significant results, only the cut off linked to the lowest *p*-value was integrated into the multivariate analysis. The analysis was performed using SPSS for Windows (version 20.0; SPSS Inc., Chicago, IL, USA).

## 3. Results

### 3.1. Patient Demographics

A total of 173 patients were included in this analytical study. The characteristics of the patients are comprehensively presented in Table 1. The median age at the time of diagnosis stood at 56 years, ranging from 27 to 85 years, while the median duration of follow-up was 36 months, spanning from 3 to 151 months.

### 3.2. Treatment Aspects

All patients underwent concurrent CRT with weekly administration of Cisplatin. Detailed treatment-related characteristics can be found in Table 1. For patients with positive lymph nodes (57 cases), an additional dose was administered either sequentially or simultaneously, resulting in a median total dose of 57.5 Gy (ranging from 52.5 to 61.0 Gy). BRT was administered to all patients, with a median dose of 37 Gy for pulsed-dose-rate BRT (ranging from 23 to 39 Gy) and 28 Gy for high-dose-rate BRT (ranging from 4 to 42 Gy).

### 3.3. Univariate Analysis

#### 3.3.1. Patient-Related Parameters

**Figure 1:**
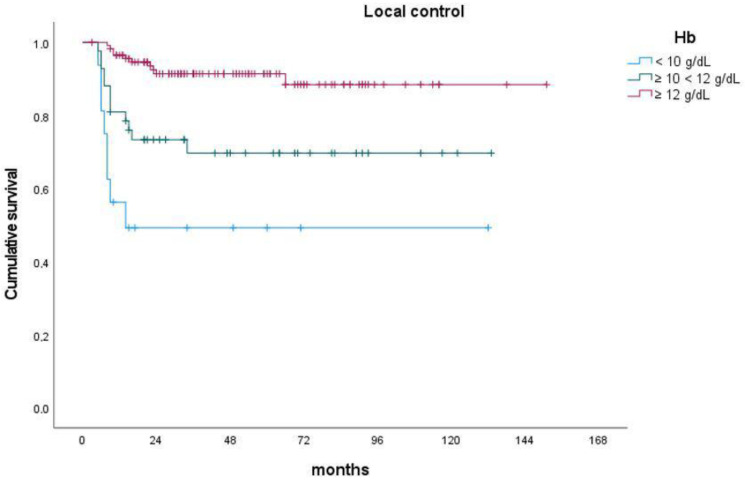
Impact of pretreatment hemoglobin level on actuarial local control.

**Figure 2:**
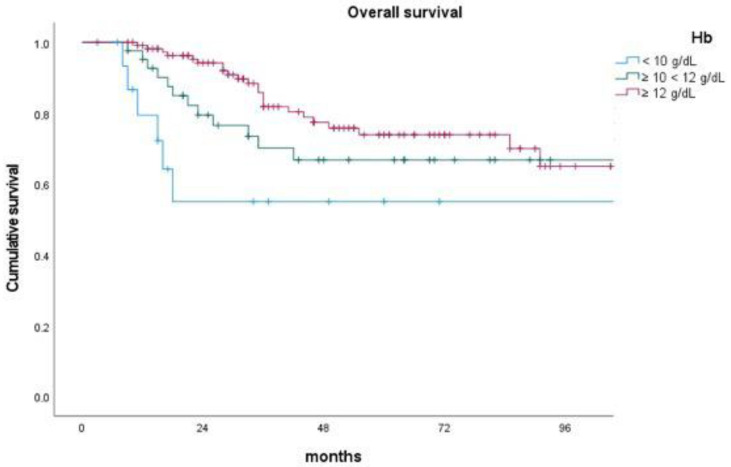
Impact of pretreatment hemoglobin level on overall survival.

In the context of patient-related factors, advanced age exhibited a significant association with poorer DMFS (*p*-value = 0.049) and OS (*p*-value = 0.003). Furthermore, lower levels of pretreatment Hb were significantly linked to inferior LC (*p*-value < 0.001) (Figure 1), DFS (*p*-value = 0.007), and OS (*p*-value = 0.040) (Figure 2).

#### 3.3.2. Tumor-Related Parameters

Our analysis showed significant correlations as follows: patients with lymph node metastases experienced worse DMFS (*p*-value = 0.045), and individuals with more advanced FIGO stages exhibited worse LC (*p*-value = 0.005), DMFS (*p*-value = 0.021), DFS (*p*-value = 0.003), and OS (*p*-value = 0.032). However, no significant differences were observed concerning maximum tumor diameter and histological type.

#### 3.3.3. Treatment-Related Parameters

Concerning treatment factors, higher total RT doses were significantly associated with improved OS (*p*-value = 0.012), while no significant variations were noted based on treatment duration (Table 2).

#### 3.3.4. Pretreatment Inflammatory Indices

In the univariate analysis, no significant correlations between the IIs and DMFS, DFS, and OS were observed. In relation to LC, significant improvements were associated with NLR values ≤ 1.6 (*p*-value = 0.022), ≤3.0 (*p*-value = 0.034), and ≤3.59 (*p*-value = 0.014). Similarly, higher LC rates were significantly linked to PLR values ≤210.00 (*p*-value = 0.017), but also with APRI values > 0.18 (*p*-value = 0.012), ANRI values > 3.47 (*p*-value = 0.044), and COP-NLR values ≤ 1 (*p*-value = 0.010). Conversely, only a trend was observed between superior LC rates and patients with SII ≤ 1000 (*p*-value = 0.077), MLR ≤ 0.26 (*p*-value = 0.100), LLR ≤ 4.17 (*p*-value = 0.088), or ALRI ≤ 9.62 (*p*-value = 0.117) (Table 3).

#### 3.3.5. Post-Treatment Inflammatory Indices

The sole statistically significant correlations were the most favorable LC in patients with NLR < 5.66 (*p*-value = 0.037) and the most favorable DMFS rate in patients with a systemic inflammatory response index (SIRI) < 3.50 (*p*-value = 0.018) (Supplementary Table S1).

#### 3.3.6. Delta Indices

The dynamic assessment of the IIs did not exhibit any significant correlation with the considered outcomes (Supplementary Table S2).

### 3.4. Multivariate Analysis

An initial multivariate analysis was performed, including all IIs with significant correlations with LC while selecting cut-off values associated with lower *p*-values from the preceding univariate analysis. We also decided to include in the multivariate Cox’s regression analysis those IIs that showed a trend for significance (a *p*-value < 0.1). This assessment confirmed a higher LC rate in patients with APRI > 0.18 (HR: 0.412; 95% CI: 0.174–0.976; *p*-value = 0.044). It is noteworthy that no substantial correlations were observed with DMFS, DFS, and OS. Only NLR > 3.59 revealed a trend for lower LC rates (HR: 1.990; 95% CI: 0.957–4.140; *p*-value = 0.065) (Table 4).

Subsequently, a secondary multivariate analysis was conducted, incorporating the clinical parameters with statistically significant correlations with at least one of the endpoints. In this analysis, no significant impact of IIs on any of the endpoints was recorded. Conversely, this analysis confirmed the strong positive influence of normal Hb levels on LC (HR: 0.141; 95% CI: 0.054–0.367; *p*-value < 0.001), DFS (HR: 0.278; 95% CI: 0.123–0.628; *p*-value = 0.002), and OS (HR: 0.255; 95% CI: 0.098–0.666; *p*-value = 0.005). Similarly, FIGO stage IV patients showed worse LC (HR: 3.271; 95% CI: 1.154–9.269; *p*-value = 0.026), DMFS (HR: 2.924; 95% CI: 1.115–7.670, *p*-value = 0.029), DFS (HR: 3.256; 95% CI: 1.527–6.943; *p*-value = 0.002), and a trend for worse OS (HR: 2.575; 95% CI: 0.914–7.250; *p*-value = 0.073) if compared with the early stages (I–II). Also, older age (≥70 years) was significantly correlated with worse DMFS (HR: 2.919; 95% CI: 1.334–6.387, *p*-value = 0.007), DFS (HR: 2.253; 95% CI: 1.122–4.526, *p*-value = 0.022), and OS (HR: 4.969; 95% CI: 2.168–11.386, *p*-value < 0.001) rates. Notably, a dose > 75 Gy is associated with significantly improved OS (HR: 0.375; 95% CI: 0.168–0.833, *p*-value = 0.016), without significant correlation with the other outcomes examined (Table 5).

## 4. Discussion

We conducted a validation study to assess whether IIs and their associated cutoff values could serve as indicators of prognosis in LACC. Our study included an analysis of patient, tumor, and treatment-related clinical parameters. The univariate and multivariate analyses confirmed that established clinical factors, such as age, FIGO stage, Hb levels, and RT dose, influence clinical outcomes [31]. The univariate analysis also showed a potential impact, primarily on LC, of IIs, like NLR, PLR, APRI, ANRI, and COP-NLR. The multivariate analysis including only IIs showed the significance of APRI in predicting LC. However, our examination of post-treatment IIs showed a correlation only between SIRI with DMFS and DFS. This discrepancy suggests that the prognostic relevance of different IIs might vary based on their evaluation either before or after treatment, as previously reported [15].

Furthermore, the lack of correlations between Delta-IIs and outcomes suggests the limited utility of the dynamical assessment of IIs over time. Moreover, the primary clinical interest resides in pretreatment IIs values due to their potential to guide personalized treatment adjustments. Conversely, after CRT and BRT, there is no evidence to support additional treatments, particularly CHT, to improve clinical outcomes [32,33].

The results of this analysis diverge from our prior study [30], in which a multivariate analysis indicated a notable correlation between higher SII values and poorer DMFS. In contrast, our present multivariate analysis reveals a significant association between APRI and LC. This discrepancy can likely be attributed to differing analytical approaches in the two studies.

In fact, in our prior study, the primary objective was to identify which IIs warranted further scrutiny. Consequently, we assessed IIs as continuous variables in statistical analyses. Conversely, our current analysis is focused on external validation of IIs and their established cut-off values from the scientific literature. As such, we evaluated IIs as dichotomized data, precisely aligned with the published thresholds.

The outcomes of our analysis stand in contrast to those reported in other studies, demonstrating a significant impact of IIs on tumor response [25], DFS [13,15,17,18,19,22,26,27], and OS [15,16,17,18,19,20,22,24,26,27]. This prompts several considerations. First, it is worth noting that other investigations have also yielded results where IIs did not significantly influence clinical outcomes [11,12,14,23]. Furthermore, we cannot exclude the possibility of publication bias or other bias (selection bias, information bias, or confounding factors) due to the retrospective nature of all these studies.

Moreover, our analysis distinguishes itself by comprehensively addressing a broader spectrum of potential confounding factors compared to prior studies (Table 6). In fact, certain analyses omitted the consideration of important factors, such as the FIGO stage [17,26] or nodal stage [13,16,22,23,25]. Additionally, treatment-related variables were frequently omitted in many studies [12,14,15,17,19,20,22,23,24,25,26]. Notably, only our study, and the paper of Koulis et al. [10], integrated Hb levels into the analysis. Interestingly, even in their analysis, no significant correlation was observed between the chosen II (NLR) and the outcome of interest, and only anemia emerged as a factor significantly associated with lower OS rates in the multivariable analysis.

On the basis of these considerations, we hypothesize that, compared to other analyses, our study has a lower risk of confounding bias. In fact, when confounding factors are not adequately controlled for in the study design or analysis, they can distort the observed relationship between the independent and dependent variables. This bias can either exaggerate or mask a real effect.

Our study has limitations that warrant acknowledgment. Certain parameters were not included in our analysis. For instance, squamous cell carcinoma antigen data, with known prognostic significance [34,35], were not incorporated due to their unavailability in most of our patient cohort. Moreover, certain other IIs, such as platelet-to-neutrophil ratio, monocyte-to-neutrophil ratio, platelet-to-white blood cell ratio, platelet-to-monocyte ratio, lymphocyte-to-monocyte ratio, eosinophil-to-lymphocyte ratio, and eosinophil-to-monocyte ratio, were not assessed in our analysis [36]. On the other hand, it was our opinion that the most common IIs, and especially those most correlated with prognosis in previous studies, were included in our analysis, and that, therefore, it was preferable not to further burden our evaluation.

Additionally, the retrospective nature of our study and lack of preliminary power analysis introduces potential limitations to the precision of outcome evaluations. However, it is important to underscore the strengths of our study, including the substantial number of cases examined and the comprehensive analysis of a wide array of clinical parameters. Furthermore, our study offers potential utility by validating results already published within the scientific community.

## 5. Conclusions

In summary, our analysis, along with other studies, presents conflicting outcomes that currently do not support the routine use of IIs as prognostic tools in patients with LACC. To enhance the clarity and reliability of future investigations, a comprehensive inclusion of potential confounding variables is needed. Based on our findings and those of Koulis et al. [10], the consideration of Hb cannot be overlooked, and that the correction of anemia is a key element in LACC patients treated with CRT.

Furthermore, advanced statistical methodologies and collaborative efforts could enhance the accuracy of prognostic models. Constructing large databases through cooperative initiatives can provide a robust foundation for developing predictive models. Notably, comprehensive evaluations encompassing multiple IIs may prove more effective in prognostication than the analysis of individual parameters. As illustrated by the study by Lee et al. [14], the combined assessment of pretreatment NLR and PLR values demonstrated a stronger association with worse OS.

## Figures and Tables

**Table 1:** Patient characteristics (adapted from Ferioli et al., 2023 [30]).

Patients n (%)	173 (100%)
Median age (range), years	56 (27–85)
Histological type, number of patients (%)	
*Squamous cell carcinoma*	173 (85.0)
*Adenocarcinoma*	26 (15.0)
Federation of Gynecology and Obstetrics stage, number of patients (%)	
*IB*	1 (0.6)
*IIA*	3 (1.7)
*IIB*	73 (42.2)
*IIIA*	9 (5.2)
*IIIB*	3 (1.7)
*IIIC1*	39 (22.5)
*IIIC2*	22 (12.7)
*IVA*	23 (13.3)
Radiotherapy technique, number of patients (%)	
*3-D conformal radiotherapy*	87 (50.3)
*Intensity-modulated radiotherapy*	66 (38.1)
*Volumetric modulated arc therapy*	20 (11.6)
Median radiotherapy dose (range), Gy	
*Prophylactic pelvic nodes irradiation*	46.0 (26.0–50.4)
*Metastatic nodes*	57.5 (52.5–61.0)
*Brachytherapy boost*	28.0 (4.0–42.0)

**Table 2:** Univariate analysis of patients, tumors, and treatment characteristics; survival outcomes are expressed in percentages.

Variable	Value	Patients No	2-y LC	5-y LC	*p*-Value	2-y DMFS	5-y DMFS	*p*-Value	2-y DFS	5-y DFS	*p*-Value	2-y OS	5-y OS	*p*-Value
Age (years)	<55	77	84.2	82.3	0.909	84.7	83.1	**0.049**	70.3	66.7	0.418	90.2	79.3	**0.003**
	55 ≤ age < 70	62	81.1	81.1		82.2	72.6		73.0	66.1		87.7	69.7	
	≥70	34	83.7	83.7		64.8	60.5		59.5	55.2		79.2	49.4	
cN	0	102	86.9	85.3	0.271	86.1	81.6	**0.045**	75.0	68.9	0.101	87.2	75.1	0.194
	1–2	71	77.3	77.3		70.8	64.7		61.0	57.1		87.5	64.6	
Total dose (Gy)	≤75	129	81.1	81.1	0.317	79.1	73.4	0.899	67.7	62.0	0.596	83.9	66.6	**0.012**
	>75	44	88.4	85.8		81.8	77.0		72.7	68.0		97.4	81.6	
FIGO stage	I–II	77	93.4	91.5	**0.005**	90.3	85.1	**0.021**	82.2	74.8	**0.003**	93.3	80.5	**0.032**
	III	73	77.7	77.7		71.1	63.8		61.2	56.6		84.4	58.8	
	IV	23	64.2	64.2		72.8	72.8		49.7	49.7		74.7	68.5	
Maximum tumor diameter	≤4	55	91.9	89.0	0.114	84,3	75.9	0.910	77.0	68.4	0.403	88.3	74,3	0.675
	>4	118	78.8	78.8		77.8	74.2		65.5	62.0		87.0	69.1	
Histologic type	SCC	139	82.8	81.6	0.598	80.0	74.4	0.799	69.1	63.6	0.917	89.0	72.3	0.305
	N-SCC	34	84.4	84.4		80.0	76.6		69.6	66.4		81.6	63.9	
Overall treatment time	<54	92	83.4	81.7	0.888	78.1	73.1	0.536	68.4	63.7	0.892	86.1	75.9	0.254
	≥54	81	82.8	82.8		82.0	76.6		70.0	64.5		88.8	65.5	
Hemoglobin	<10	16	49.2	49.2	**<0.001**	72.3	72.3	0.270	48.1	48.1	**0.007**	55.0	55.0	**0.040**
	10 ≤ Hb < 12	42	73.3	69.7		79.5	79.5		63.4	60.1		79.5	66.8	
	≥12	115	91.4	91.4		81.4	73.9		74.3	67.7		94.2	73.9	

Legend: cN: clinical nodal stage; DFS: disease-free survival; DMFS: distant metastasis-free survival; FIGO: International Federation of Gynecology and Obstetrics; LC: local control; OS: overall survival.

**Table 3:** Univariate analysis of inflammatory indices (pretreatment values); survival outcomes are expressed in percentages.

Variable	Value #	Patients No	2-y LC	5-y LC	*p*-Value	2-y DMFS	5-y DMFS	*p*-Value	2-y DFS	5-y DFS	*p*-Value	2-y OS	5-y OS	*p*-Value
NLR	≤1.60	24	100.0	100.0	**0.022**	90.9	83.3	0.243	78.9	78.9	0.075	91.3	72.9	0.572
	>1.60	149	84.3	79.4		79.0	73.2		66.4	61.7		84.8	68.6	
NLR	≤2.00	46	87.5	87.5	0.209	77.4	73.1	0.628	67.5	63.5	0.918	85.3	66.2	0.707
	>2.00	127	81.4	80.2		81.7	75.1		69.7	64.2		85.9	70.4	
NLR	≤2.50	73	87.9	87.9	0.145	80.4	74.0	0.698	70.7	66.5	0.671	87.9	70.1	0.524
	>2.50	100	79.5	78.0		80.8	75.0		68.0	62.2		84.2	68.8	
NLR	≤3.00	99	88.8	87.0	**0.034**	81.2	73.7	0.804	69.9	62.1	0.958	89.9	67.4	0.389
	>3.00	74	75.4	75.4		79.9	75.9		68.2	66.2		80.3	70.9	
NLR	≤3.59	122	87.6	86.2	**0.014**	81.6	74.6	0.698	71.6	65.5	0.255	90.3	69.5	0.164
	>3.59	51	72.3	72.3		78.5	75.1		63.4	60.4		74.8	68.2	
NLR	≤3.80	125	86.2	84.8	0.079	80.9	74.0	0.826	70.5	64.5	0.510	88.7	68.3	0.479
	>3.80	48	74.9	74.9		80.2	76.1		65.6	62.5		78.2	71.3	
NLR	≤5.00	142	90.0	83.9	0.129	80.2	74.1	0.581	70.4	65.2	0.482	87.8	67.6	0.571
	>5.00	31	74.1	74.1		83.0	77.8		63.0	58.5		76.1	76.1	
PLR	≤3.85	2	100	100	0.595	100	100	0.596	100	100	0.482	100	100	0.671
	>3.85	171	82.9	82.0		80.5	74.5		68.9	63.8		85.6	69.2	
PLR	≤70.00	7	85.7	85.7	0.957	100.0	100.0	0.267	85.7	85.7	0.502	80.0	80.0	0.982
	>70.00	166	83.0	82.1		80.0	74.0		68.7	63.5		85.9	69.2	
PLR	≤133.02	63	87.9	84.2	0.479	83.9	72.0	0.527	73.0	61.5	0.943	91.4	60.3	0.657
	>133.02	110	80.3	80.3		78.8	75.2		67.0	64.4		82.7	72.4	
PLR	≤136.6	67	88.6	85.2	0.334	84.8	73.4	0360	74.5	63.5	0.644	91.8	62.3	0.854
	>136.6	106	79.6	79.6		78.1	74.4		65.9	63.3		82.2	71.7	
PLR	≤139.2	69	88.9	85.6	0.268	85.3	74.2	0.342	73.8	62.9	0.736	92.1	63.3	0.772
	>139.2	104	79.2	79.2		77.7	73.9		66.3	63.7		81.8	72.1	
PLR	≤148.8	83	89.6	87.2	0.097	84.8	76.2	0.165	75.5	67.0	0.354	90.8	65.4	0.688
	>148.8	90	77.2	77.2		76.8	72.5		63.5	60.7		81.3	72.1	
PLR	≤154.17	88	88.7	86.5	0.100	93.1	75.2	0.269	74.5	66.6	0.373	88.6	65.1	0.595
	>154.17	85	77.2	77.2		77.9	73.4		63.8	60.8		82.8	72.0	
PLR	≤158.00	90	89.0	86.9	0.073	83.6	75.9	0.201	75.1	67.5	0.266	88.9	66.2	0.733
	>158.00	83	76.6	76.6		77.3	72.7		62.9	59.9		82.3	71.4	
PLR	≤172.50	103	86.2	84.4	0.245	83.9	76.3	0.203	72.0	65.9	0.408	88.1	67.9	0.619
	>172.50	70	78.3	78.3		75.7	71.9		64.8	61.1		82.2	70.7	
PLR	≤210.00	127	88.3	85.9	**0.017**	83.1	77.3	0.147	72.7	66.7	0.185	88.8	73.6	0.080
	>210.00	46	71.4	71.4		73.6	67.5		59.2	56.4		77.4	63.3	
SII	≤1000.00	106	87.8	86.1	0.077	80.3	74.3	0.864	70.8	63.3	0.759	87.6	67.9	0.734
	>1000.00	67	75.6	75.6		81.2	75.0		66.4	64.2		82.8	70.7	
LLR	≤4.17	93	88.1	86.2	0.088	79.2	75.4	0.601	69.0	60.9	0.714	81.9	39.2	0.328
	>4.17	80	77.2	77.2		82.1	73.8		69.2	67.3		89.7	69.4	
LLR	≤5.28	127	85.5	84.2	0.138	80.6	74.7	0.687	70.9	65.0	0.324	83.4	68.4	0.259
	>5.28	46	76.0	76.0		80.7	74.3		64.2	61.0		91.4	71.7	
MLR	≤0.26	103	87.1	85.4	0.100	79.7	72.8	0.347	68.1	63.9	0.779	88.2	66.1	0.791
	>0.26	70	77.0	77.0		82.0	77.4		70.6	64.2		82.2	74.1	
MLR	≤0.33	122	89.9	84.9	0.268	80.3	74.4	0.713	67.2	62.4	0.517	87.4	66.6	0.702
	>0.33	51	78.4	78.4		81.4	75.3		73.9	68.1		81.7	76.3	
APRI	≤0.18	96	76.4	76.4	**0.012**	80.1	77.4	0.769	64.7	64.7	0.325	84.4	70.7	0.327
	>0.18	77	91.5	89.1		79.6	71.3		74.7	62.6		91.0	70.2	
ALRI	≤9.62	87	87.4	87.4	0.117	81.2	77.6	0.950	71.9	70.1	0.380	89.6	72.9	0.848
	>9.62	86	78.6	77.0		78.6	72.1		66.3	58.6		85.0	68.6	
ANRI	≤3.47	87	77.6	77.6	**0.044**	81.5	78.2	0.822	66.8	65.3	0.462	84.0	69.9	0.186
	>3.47	86	88.6	88.6		78.3	71.6		71.5	62.8		90.6	71.2	
COP-NLR *	≤1	137	86.7	85.5	**0.010**	80.2	74.5	0.733	70.6	63.9	0.390	90.3	70.0	0.250
	>1	36	69.0	69.0		79.0	75.2		63.0	63.0		75.6	71.6	

Legend: ALRI: aspartate aminotransferase-to-lymphocyte ratio index; ANRI: aspartate transaminase-to-neutrophil ratio index; APRI: aspartate aminotransferase/platelet count ratio index; COP-NLR: combination of platelet count and neutrophil-to-lymphocyte ratio; DMFS: distant metastasis-free survival; DFS: disease-free survival; LC: local control; LLR: leukocyte-to-lymphocyte ratio; MLR: monocyte-to-lymphocyte ratio; NLR: neutrophil-to-lymphocyte ratio; OS: overall survival; PFS: progression-free survival; PLR: platelet-to-lymphocyte ratio; SII: systemic immune inflammation index; SIRI: systemic inflammatory response index; * COP-NLR scored as follows: 0: NLR < 3 and PLT < 300; 1: NLR > 3 or PLT > 300; 2: NLR > 3 and PLT > 300. #: different cut off from published studies.

**Table 4:** Multivariate Cox’s analysis of inflammatory indices (pre-treatment values); survival outcomes are expresses in percentages. Only statistically significant values (and values with trend for significance) are shown.

Parameter	Values	Patients N (%)	LC			DMFS			DFS			OS		
			HR	95% CI	*p*-Value	HR	95% CI	*p*-Value	HR	95% CI	*p*-Value	HR	95% CI	*p*-Value
NLR	≤3.59	122	1	rif.					1	rif.				
	>3.59	51	1.990	0.957–4.140	0.065				1.360	0.795–2.328	0.262			
PLR	≤210.00	127				1	rif.					1	rif.	
	>210.00	46				1.360	0.706–2.620	0.357				1.646	0.885–3.059	0.115
APRI	≤0.18	96	1	rif.										
	>0.18	77	0.412	0.174–0.976	**0.044**									

Legend: APRI: aspartate aminotransferase/platelet count ratio index; DFS: disease free survival; DMFS: distant metastasis free survival; FIGO: International Federation of Gynecology and Obstetrics; LC: local control; NLR: neutrophil to lymphocyte ratio; OS: overall survival; PLR: platelet to lymphocyte ratio.

**Table 5:** Multivariate Cox’s analysis of inflammatory indices (pre-treatment values) and clinical parameters. Only statistically significant values (and values with trend for significance) are shown.

Parameter	Values	Patients N (%)	LC			DMFS			DFS			OS		
			HR	95% CI	*p*-Value	HR	95% CI	*p*-Value	HR	95% CI	*p*-Value	HR	95% CI	*p*-Value
Age (years)	<55	77				1	rif.	**0.025**	1	rif.	**0.061**	1	rif.	**<0.001**
	55 ≤ age < 70	62				1.378	0.668–2.839	0.385	1.141	0.621–2.095	0.671	2.014	0.929–4.366	0.076
	≥70	34				2.919	1.334–6.387	**0.007**	2.253	1.122–4.526	**0.022**	4.969	2.168–11.386	**<0.001**
Total dose (Gy)	≤75	129										1	rif.	
	>75	44										0.375	0.168–0.833	**0.016**
FIGO stage	I–II	77	1	rif.	0.077	1	rif.	**0.015**	1	rif.	**0.004**	1	rif.	**0.005**
	III	73	2.164	0.877–5.339	0.094	2.656	1.315–5.364	**0.006**	2.203	1.222–3.972	**0.009**	3.220	1.589–6.526	**0.001**
	IV	23	3.271	1.154–9.269	**0.026**	2.924	1.115–7.670	**0.029**	3.256	1.527–6.943	**0.002**	2.575	0.914–7.250	0.073
Hemoglobin	<10	16	1	rif.	**<0.001**				1	rif.	**0.008**	1	rif.	**0.013**
	10 ≤ x <12	42	0.467	0.188–1.157	0.100				0.445	0.193–1.027	0.058	0.488	0.184–1.295	0.150
	≥12	115	0.141	0.054–0.367	**<0.001**				0.278	0.123–0.628	**0.002**	0.255	0.098–0.666	**0.005**
LLR	≤4.17	93							1	rif.				
	>4.17	80							0.628	0.363–1.086	0.096			

Legend: APRI: aspartate aminotransferase/platelet count ratio index; DFS: disease free survival; DMFS: distant metastasis free survival; FIGO: International Federation of Gynecology and Obstetrics; LC: local control; LLR: leukocyte-to-lymphocyte ratio; NLR: neutrophil to lymphocyte ratio; OS: overall survival; PLR: platelet to lymphocyte ratio.

**Table 6:** Comparison between the results of previous analyses and those of our series (adapted from Ferioli et al., 2023 [30]).

Author/Year	Evaluated Indexes	Cut-Off	Outcome Predictions	Confounders Considered
Koulis et al./2017 [10]	NLR	5 11.5	<PFS and <OS if Hb < 11.5; no impact of NLR alone (pre-CRT)	age; anemia; histological type; FIGO; T size; N stage; treatment
Haraga et al./2016 [12]	NLR PLR PNI	2.85 172.5 48.5	<OS and <PFS if <PNI; no impact of NLR and PLR (pre-CRT)	histological type; FIGO; T size; N stage; lymphovascular invasion
Jeong et al./2019 [13]	NLR	2.8	< PFS if >NLR; no impact on OS	age; histological type; T size; FIGO; treatment
Lee et al./2021 [14]	NLR PLR	2.34 148.9	<OS only if both >NLR and >PLR	age; histological type; FIGO; T size; N stage
Lee et al./2020 [15]	NLR MLR PLR	3.04 174.3 3.85	<DFS if >NLR, >ΔNLR, >ΔPLR (post-CRT); <OS if >NLR (post-CRT); no impact on OS of NLR, MLR, PLR (pre-CRT), ΔNLR, ΔMLR, ΔPLR, and MLR, PLR (post-CRT)	age; histological type; FIGO; T size; N stage
Lee et al./2012 [16]	NLR	1.9	<OS if >NLR (pre-CRT)	age; histological type; FIGO; treatment
Li et al./2021 [17]	NLR PLR MLR SIRI BLR	2.49 154.2 0.26 1.02 0.02	<OS and <PFS if >NLR and >MLR (pre-CRT); no impact of PLR, BLR, SIRI (pre-CRT)	age; histological type; T size; N stage; menopausal status
Mizunuma et al./2015 [18]	NLR	2.5	<OS and <PFS if >NLR (pre-CRT)	age; histological type; FIGO; T size; N stage; treatment
Onal et al./2016 [19]	NLR PLR	3.03 133.0	<OS, <PFS if >NLR; no impact of PLR (pre-CRT)	age; histological type; FIGO; T size; N stage
Wang et al./2016 [20]	NLR	2	<OS if >NLR (pre-CRT)	age; histological type; FIGO; T size; N stage
Holub et al./2019 [22]	NLR PLR SII ELR	3.8 210 1000 0.07	>OS if >ELR; <PFS if >PLR or >SII (pre-CRT)	age; histological type; FIGO; HPV status
Kim et al./2020 [23]	NLR PLR LMR	2.33 136.6 4.17	<PFS and OS if >ΔNLR; no impact of NLR, PLR, LMR (pre-CRT), and of ΔPLR, ΔLMR	age; histological type; FIGO
Jonska-Gymrec et al./2018 [24]	NLR PLR MLR	1.6 158 0.33	<OS if> NLR; no impact of PLR (pre-CRT)	age; histological type; FIGO; T grade; N stage
Chauan et al./2022 [25]	NLR PLR	3 70	>CR rate if <NLR and <PLR	age; histological type; FIGO
Li et al./2016 [26]	LMR	5.28	>PFS and >OS if >LMR (pre-CRT)	age; histological type; N stage; HPV status
Liang et al./2022 [27]	NLR	3.87	<OS and <PFS if >NLR (pre-CRT)	age; BMI; histological type; FIGO; T size; N stage; treatment
Ferioli et al./2023 [30]	NLR, PLR, MLR, SII, LLR, APRI, ALRI, SIRI, ANRI, COP *	c.v.	<distant metastasis-free survival if >SII	age; BMI; anemia; histological type; FIGO; T size; N stage; treatment; PNI
Present series	NLR, PLR, MLR, SII, LLR, APRI, ALRI, SIRI, ANRI, COP *	#	>LC if <NLR and >LLR (only without including clinical parameters in the multivariate analysis)	age; anemia; histological type; FIGO; T size; N stage; treatment

Legend: ALRI: aspartate aminotransferase-to-lymphocyte ratio index; ANRI: aspartate transaminase-to-neutrophil ratio index; APRI: aspartate aminotransferase/platelet count ratio index; BLR: basophil/lymphocyte ratio; BMI: body mass index; cN+: clinical positive nodes; COP-NLR: combination of platelet count and neutrophil-to-lymphocyte ratio; CR: complete response; CRT: chemoradiation; DFS: disease-free survival; ELR: eosinophils–lymphocyte ratio; FIGO: International Federation of Gynecology and Obstetrics; Hb: hemoglobin; LLR: leukocyte-to-lymphocyte ratio; MLR: monocyte-to-lymphocyte ratio; N: nodal; NLR: neutrophil-to-lymphocyte ratio; OS: overall survival; PFS: progression-free survival; PLR: platelet-to-lymphocyte ratio; RT: radiotherapy; SII: systemic immune inflammation index; SIRI: systemic inflammatory response index; T: tumor; * COP-NLR scored as follows: 0: NLR < 3 and PLT < 300; 1: NLR > 3 or PLT > 300; 2: NLR > 3 and PLT > 300. #: different cut off from published studies.

## Data Availability

Data supporting the reported results will be made available upon reasonable request.

## Supplementary Materials

The following supporting information can be downloaded at: https://www.mdpi.com/article/10.3390/cancers15205056/s1, Table S1: univariate analysis of inflammatory indices (post-treatment values); survival outcomes are expressed in percentages; Table S2: univariate analysis of Delta inflammatory indices (post-treatment values minus pretreatment values); survival outcomes are expressed in percentages.
