# Supplementary material for: Decoding the Complexity of Systemic Inflammation Predictors in Locally Advanced Cervical Cancer, with Hemoglobin as the Hidden Key (the ESTHER Study)
Source: Cancers (Basel). 2023 Oct 19;15(20):5056. doi: 10.3390/cancers15205056 (PMC10605166; doi:10.3390/cancers15205056)
Supplement: Supplementary file 1 [file cancers-15-05056-s001.zip › cancers-2611188-supplementary.pdf]

Table S1: univariate analysis of inflammatory indices (post-treatment values); survival outcomes are expresses in percentages.

Variable	Value	2-y LC	5-y LC	p	2-y DMFS	5-y DMFS	p	2-y DFS	5-y DFS	P	2-y OS	5-y OS	P
NLR	< 5.665	88.7	88.7	**0.037**	76.5	73.5	0.856	71.9	70.5	0.065	92.0	74.3	0.197
	≥ 5.665	78.0	76.2		83.2	75.4		66.7	57.5		83.2	67.4	
PLR	< 465.135	85.7	85.7	0.440	77.1	71.4	0.378	67.7	64.0	0.786	90.3	67.9	0.538
	≥ 465.135	80.7	79.1		82.3	77.5		70.4	64.0		84.8	72.4	
MLR	< 0.685	84.6	84.6	0.644	78.3	73.1	0.777	70.4	67.2	0.556	87.8	71.4	0.712
	≥ 0.685	81.6	79.9		81.3	76.1		68.0	61.0		86.9	70.0	
SII	< 977.455	87.4	87.4	0.105	77.6	72.2	0.765	69.5	66.1	0.605	90.6	69.9	0.855
	≥ 977.455	79.2	77.6		82.1	77.0		68.8	62.1		84.6	71.1	
LLR	< 8	87.3	87.3	0.093	76.5	70.1	0.576	69.5	66.3	0.356	92.0	70.3	0.679
	≥ 8	79.2	77.5		83.2	79.2		68.9	61.8		83.2	71.2	
APRI	< 21.34	80.7	80.7	0.467	78.6	76.5	0.701	67.2	63.0	0.523	83.1	69.8	0.699
	≥ 21.34	84.8	83.2		80.9	73.5		70.6	64.8		90.8	71.5	
ALRI	< 47.06	84.5	84.5	0.715	78.4	73.0	0.426	70.7	67.3	0.653	89.5	70.1	0.762
	≥ 47.06	81.9	80.3		80.9	76.1		67.8	61.7		85.7	70.9	
SIRI	< 3.505	87.6	85.6	0.159	85.6	82.1	**0.018**	76.2	70.3	**0.044**	90.9	72.1	0.462
	≥ 3.505	78.8	78.8		74.3	68.3		62.7	58.2		83.9	68.9	
ANRI	< 7.48	80.5	80.5	0.418	78.3	76.1	0.602	66.7	62.4	0.436	82.9	69.2	0.602
	≥ 7.48	85.0	83.4		81.1	73.9		70.9	65.2		90.9	71.9	
COP-NLR *	< 1	81.4	81.4	1	73.0	63.8	0.328	66.6	62.4	0.979	1	60.0	0.439
	≥ 1	83.4	82.2		81.3	76.8		69.7	64.3		85.0	73.1	

Legend: ALRI: aspartate aminotransferase to lymphocyte ratio index; ANRI: aspartate transaminase to neutrophil ratio index; APRI: aspartate aminotransferase/platelet count ratio index; COP-NLR: combination of platelet count and neutrophil to lymphocyte ratio; DFS: disease free survival; DMFS: distant metastasis free survival; LC: local control; LLR: leukocyte-to-lymphocyte ratio; MLR: monocyte to lymphocyte ratio; NLR: neutrophil to lymphocyte ratio; OS: overall survival; PLR: platelet to lymphocyte ratio; SII: systemic immune inflammation index; SIRI: systemic inflammatory response index; * COP-NLR scored as follows: 0: NLR<3 and PLT<300; 1: NLR>3 or PLT>300; 2: NLR>3 and PLT>300.

Table S2: univariate analysis of Delta inflammatory indices (post-treatment values *minus* pre-treatment values); survival outcomes are expresses in percentages.

Variable	Value	2-y LC	5-y LC	p	2-y DMFS	5-y DMFS	p	2-y DFS	5-y DFS	p	2-y OS	5-y OS	P
Delta NLR	< 2.79	84.9	84.9	0.378	76.5	71.9	0.799	68.1	66.7	0.367	89.4	73.2	0.529
	≥ 2.79	81.5	79.8		83.1	76.9		70.2	61.2		85.5	68.3	
Delta PLR	< 295.58	82.8	82.8	0.869	79.0	73.1	0.889	67.0	63.4	0.704	90.0	69.4	0.543
	≥ 295.58	83.3	81.7		80.7	75.9		71.0	64.4		85.3	71.4	
Delta MLR	< 0.42	83.3	83.3	0.701	76.5	71.3	0.892	67.9	64.6	0.659	87.6	72.1	0.727
	≥ 0.42	82.8	81.1		82.7	77.5		70.3	63.3		87.1	69.4	
Delta SII	< 86.66	80.0	80.0	0.557	74.6	70.0	0.556	64.8	62.7	0.866	88.4	69.5	0.836
	≥ 86.66	84.5	83.1		82.4	76.8		71.2	64.3		86.9	71.2	
Delta LLR	< 3.58	86.0	86.0	0.225	76.3	71.6	0.770	67.8	66.4	0.437	91.8	73.2	0.464
	≥ 3.58	80.5	78.9		83.2	77.3		70.4	61.7		83.4	68.4	
Delta APRI	< 20.925	80.6	80.6	0.444	78.4	76.2	0.619	66.9	62.5	0.463	82.9	69.2	0.608
	≥ 20.925	84.9	83.3		81.1	73.8		70.8	65.1		90.9	71.9	
Delta ALRI	< 37.06	84.7	84.7	0.688	77.1	73.6	0.425	68.2	66.6	0.859	89.6	71.8	0.569
	≥ 37.06	81.8	80.3		82.1	75.7		69.8	62.3		85.7	69.7	
Delta SIRI	< -16.39	84.7	82.7	0.882	85.2	79.5	0.469	69.4	65.6	0.950	87.8	74.4	0.508
	≥ -16.39	81.6	81.6		75.3	70.4		68.9	62.6		86.9	67.4	
Delta ANRI	< 3.56	78.8	78.8	0.253	76.4	74.1	0.238	63.6	56.6	0.105	83.7	60.5	0.115
	≥ 3.56	86.2	84.7		82.4	76.9		73.2	69.1		88.8	77.4	
Delta COP *	< 0	80.7	80.7	0.302	79.0	75.6	1	83.7	67.3	0.664	86.9	72.2	0.858
	≥ 0	88.0	84.7		81.9	70.7		70.8	54.4		88.2	65.9	

Legend: ALRI: aspartate aminotransferase to lymphocyte ratio index; ANRI: aspartate transaminase to neutrophil ratio index; APRI: aspartate aminotransferase/platelet count ratio index; COP-NLR: combination of platelet count and neutrophil to lymphocyte ratio; DFS: disease free survival; DMFS: distant metastasis free survival; LC: local control; LLR: leukocyte-to-lymphocyte ratio; MLR: monocyte to lymphocyte ratio; NLR: neutrophil to lymphocyte ratio; OS: overall survival; PLR: platelet to lymphocyte ratio; SII: systemic immune inflammation index; SIRI: systemic inflammatory response index; * COP scored as follows: 0: NLR<3 & PLT<300; 1: NLR>3 or PLT>300; 2: NLR>3 and PLT>300
